# Influence of hot air drying on capsaicinoids, phenolics, flavonoids and antioxidant activities of ‘Super Hot’ chilies

**DOI:** 10.7717/peerj.13423

**Published:** 2022-05-25

**Authors:** Esther Shiau Ping Yap, Apiradee Uthairatanakij, Natta Laohakunjit, Pongphen Jitareerat

**Affiliations:** 1 Division of Postharvest Technology, School of Bioresources and Technology, King Mongkut’s Institute of Technology Thonburi, Bangkok, Thailand; 2 Division of Biochemical Technology, School of Bioresources and Technology, King Mongkut’s Institute of Technology Thonburi, Bangkok, Thailand

**Keywords:** Hot air drying, Capsaicinoids, Capsicum annuum, Bioactive compounds, ‘Super Hot’ chili

## Abstract

Hot air drying is an alternative technique to either maintain or increase bioactive compounds in agricultural products because temperatures can be controlled. The effects of different hot air oven drying temperatures and times on the physicochemical changes, bioactive compounds (capsaicinoids, phenolic and flavonoid profiles and contents) and antioxidant activities in dried ‘Super Hot’ chili fruits were evaluated. The chilies were dried in a hot air oven at low (60–100 °C) or high (120–160 °C) temperatures for 30, 60, 120 min and at 12–13% moisture content (MC). The main compounds presented in chili fruits were capsaicinoids, limonene, pinene, tocopherol and oleic acid, regardless of drying temperature and time. Although the total flavonoid contents decreased during the drying process, the total phenolic contents increased (38–51%), and capsaicinoids, the primary pungent compounds, increased six-times at 120–160 °C compared to the fresh chilies. The phenolic profiles showed that chlorogenic acid was the most stable and abundant amongst the nine quantified phenolic compounds. In the flavonoid profile, both rutin and quercetin can be detected at a high temperature of 160 °C, with a decreasing trend. The main pungent compounds, capsaicin and dihydrocapsaicin, were found to increase compared to the fresh chilies, especially at 12–13% wet basis (w.b.). Although the antioxidant activities (ABTS• + and DPPH•) of dried chilies at all temperatures decreased with increasing drying time, these activities were still detected. Therefore, drying chilies at 160 °C (120 min) can not only maintain the capsaicinoids, phenolics and flavonoids that can be utilized by the pharmaceutical and food industry, but can also reduce the production time.

## Introduction

Chilies from the genus *Capsicum* are valued worldwide. They are, rich in bioactive compounds (capsaicinoids, gallic acid, caffeic acid, chlorogenic acid and rutin, among others) (Shaimaa et al., 2016). Previous reports indicate that capsaicinoids, which consist of capsaicin (C), dihydrocapsaicin (DHC), nonivamide, nordihydrocapsaicin and homocapsaicin, play a role in chilies’ pungency and possess antioxidative properties (Maokam, Techawongstien & Chanthai, 2014). However, chilies are perishable crops with high moisture content, and rapid quality changes, resulting in short shelf life and leading to postharvest and economic losses (Samira, Woldetsadik & Workneh, 2013).

Several studies have reported that phenolic compounds, capsaicinoid contents and antioxidant capacity in dried chilies either decreased (Zhou et al., 2016) or increased (Chaaban et al., 2016) in comparison to fresh chilies. For instance, Reis et al. (2013) found that drying at temperatures above 45 °C caused a loss in phenolic compounds (85%), C (34%) and DHC (53%) in red peppers. Furthermore, the capsaicinoid contents were reduced by 18% when the drying temperature was above 60 °C (Zhou et al., 2016). Conversely, Arslan & Özcan (2011) found that antioxidant capacity and 2,2-diphenyl-1-picrylhydrazyl (DPPH) free radical scavenging activity in dried chilies increased by 61–65% and 28–35%, respectively, while being compared with the same parameters in fresh chilies. An increase in C in chilies was also observed when dried by 71–92% (approximately 10–11% moisture content on a wet basis (w.b.)), depending on chili varieties (Popelka et al., 2017). According to Chaaban et al. (2016) and Deng et al. (2018), the increase of C was due to the structural transformation and the destruction of cellular structures from the heat generated by the hot air oven, causing the C to spread from the pericarp to other parts of the pepper. Moreover, volatiles during drying of chilies were also found to be either retained (*γ*-himachalene), lost (n-hexanol) or produced (furfural) compared to the volatiles in fresh chilies (Chairote & Intachum, 2016). Furthermore, Yazdizadeh Shotorbani, Jamei & Heidari (2013) found a reduction in total phenolic content (TPC) but an increase in total flavonoid and ferric reducing antioxidant power (FRAP) in *Capsicum annuum* when dried at 65 °C for 60 min compared to 30 min. In a study by Zhou et al. (2016), a decreasing trend for capsaicinoid contents was also seen; however, it was constant after 150 min in a hot air oven, depending on the drying temperature (*i.e.,* 60, 70, and 80 °C). The degradation of capsaicinoids is because of chemical changes in the structure, such as deprotonation of the hydroxyl group (Suresh, Manjunatha & Srinivasan, 2007) or oxidation reactions catalyzed by peroxidase at low drying temperatures (Topuz & Ozdemir, 2004). These authors attributed the changes in bioactive compounds during drying to the heat instability of compounds, the release of cell-bound compounds, changes in compound structure and the formation of new compounds due to heat.

The studies mentioned above generally used drying temperatures between 45 °C and 100 °C to investigate its varying effects on the bioactive compounds. However, high temperature affects the taste and odor compound formation of dried products (Harkouss et al., 2015). When drying at low temperatures, spoilage of the product may occur as it takes a long time to dry (Hawaree, Chiewchan & Devahastin, 2009). Few studies on dried chilies have been conducted using temperatures above 100 °C. Thus, using hot air drying at high temperatures (>100 °C) can be the solution to maintain the color and bioactive compounds of dried chilies as the temperatures can be controlled. Additionally, the drying process is used to reduce the MC of chilies to a level that is low enough to inhibit the growth of microorganisms that are favored with a high moisture content and extend the shelf life (Yadav & Singh, 2014; Zhou et al., 2016). This process can be accomplished by energy efficient hot air drying that utilizes heat to evaporate the MC of a commodity (Torki-Harchegani et al., 2016). However, drying depends on the drying temperature and time that influence the chemical, such as bioactive compound contents, and physical and biological properties of a commodity, especially in chilies (Shaimaa et al., 2016). Hence, the present work was undertaken to compare the physicochemical properties of chilies dried at: (a) low temperature (60 °C, 80 °C and 100 °C), long time (1440, 600 and 270 min) and (b) high temperature (120 °C, 140 °C and 160 °C), short time (210, 180 and 120 min).

To the best of our knowledge, insufficient information is available on the effects of high temperature drying using a hot air oven on the physicochemical changes, bioactive compounds in chilies. Therefore, the objectives of this study were to investigate the effects of hot air drying temperature and drying time on the color, capsaicinoids , phenolic, flavonoid profiles and contente, as well as antioxidant activities of ‘Super Hot’ chilies. In this study, the ‘Super Hot’ chili cultivar was chosen due to its high pungency and demand in Thailand. Additionally, there is a lack of studies that have determined the influence of both drying temperature and time on changes in metabolites and bioactive compounds, especially capsaicinoids, in the ‘Super Hot’ chili cultivar.

## Materials & Methods

### Plant materials, sample preparation and drying conditions

The ‘Super Hot’ red chilies (*C. annuum*) were harvested at 35–45 days after anthesis from a local farm located in Nakhon Ratchasima Province, Thailand, in July 2018. Subsequently, they were transported to King Mongkut’s University of Technology Thonburi within 2 h. This harvested batch was used to sort for fruit quality. Fruits of similar size and color, without mechanical damage (8 kg), were selected and washed, destalked and air-dried to remove excess water before drying. The color and weight were determined in fresh samples, before being frozen with liquid nitrogen and kept at −20 °C before further chemical analyses. Chilies (400 g) were placed on trays in a single layer and loaded into a hot air oven (Memmert UE200; Germany) with a temperature accuracy of ±1 °C, 100% air circulation and a volume of 26.4 L. They were dried until they reached a moisture content (MC) of 12–13% w.b. and were classified as: (a) low temperature (60 °C, 80 °C and 100 °C), long time (1440, 600 and 270 min) and (b) high temperature (120 °C, 140 °C and 160 °C), short time (210, 180 and 120 min). Samples were also taken at 30, 60, and 120 min for all drying temperatures to monitor the changes in compound levels. The end MC of 12–13% was selected according to the Thai Agricultural Standard (TAS 3001-2010) for dried chili fruits (Ministry of Agriculture and Cooperatives, 2021). The differences in end drying times for each temperature occurred due to the fact that higher temperatures reduced drying time and increased the effective moisture diffusivity (Kaleemullah & Kailappan, 2005). The color and weight of fresh and dried chili at 4 sampling times were determined, and then samples were frozen with liquid nitrogen and kept at −20 °C for further chemical analyses. Changes in the physicochemical and bioactive compounds of the dried samples at time intervals were determined as follows.

### Determination of moisture content

The moisture content (MC) was determined according to the Association of Analytical Chemists (AOAC, 2000). The moisture content was also determined in fresh and dried chili samples. Ten grams of chili samples were taken and dried for 24 h in an oven at 105 ° C. The MC was calculated in percentage on a w.b. as follows in Eq. (1):

(1) \begin{eqnarray*}\mathrm{MC}(\text{%}\mathrm{w}.\mathrm{b}.)= \frac{\text{weight of fresh sample}(\mathrm{g})-\text{weight at sampling time}(\mathrm{g})}{\text{weight of fresh sample}(\mathrm{g})} \times 100.\end{eqnarray*}

### Surface color parameters

A Minolta CR-400 colorimeter (New Jersey, USA) with an aperture size of 0.8 cm was used to determine the surface color of the samples (*L*^∗^, *a*^∗^ and *b*^∗^ values). The *L*^∗^, *a*^∗^ and *b*^∗^ values showed the lightness, redness and yellowness, respectively. The surface color of fresh and dried chilies was recorded by placing four chilies together to cover the aperture of the colorimeter to ensure that the aperture was fully covered and accurate measurements were taken (Giese, 2003). The values were recorded as the mean of three determinations at three different sample locations. Total color differences were calculated according to Eq. (2) (Sigge, Hansmann & Joubert, 2001):

(2) \begin{eqnarray*}\text{Total color differences}(\Delta E)=\sqrt{({L}^{\ast }-{L}_{o}^{\ast })^{2}+({a}^{\ast }-{a}_{o}^{\ast })^{2}+({b}^{\ast }-{b}_{o}^{\ast })^{2}}\end{eqnarray*}

where ${L}_{\mathrm{o}}^{\ast }$, ${a}_{\mathrm{o}}^{\ast }$ and ${b}_{\mathrm{o}}^{\ast }$ were the values of fresh chilies (reference values).

### Plant extract

The fresh and dried chilies were disintegrated and extracted using the method described by Maokam, Techawongstien & Chanthai (2014). Samples (2 g) were homogenized with 20 mL methanol and placed in a water bath at 60 °C for 2 h. The mixture was then centrifuged for 5 min at 3,130× g and 4 °C, filtered with Whatman No. 42, evaporated using a BÜCHI Rotavapor R-200 rotary evaporator at 40 °C to obtain the crude extract and redissolved with five mL methanol. The extracts were used to determine phenolic and flavonoid compounds, total phenolic content (TPC) and total flavonoid content (TFC) analyses.

### Determination of bioactive compounds profiling and capsaicinoid derivatives

The extraction solution was analyzed and identified for bioactive compounds profiling and capsaicinoid derivatives through gas chromatography–mass spectrometry (GC–MS) using an Agilent 7890A gas chromatograph (Agilent Technologies, Santa Clara, CA, USA). The compounds were separated using a silica capillary column HP5-MS (30 m length, 0.25 mm inner diameter (i.d.), 0.25 µm and film thickness; Agilent J&W, Santa Clara, CA, USA). Helium was used as the carrier gas at a constant flow of 2.0 mL/min. The temperatures of the injector and detector were 250 °C and 280 °C, respectively. The initial temperature condition was 35 °C for 10 min and was increased to 95 °C at 3 °C/min, then to 270 °C at a rate of 10 °C/min (constant for 10 min) and finally to 300 °C at a rate of 3 °C/min (constant for 10 min). The mass scan range used was 30–550 atomic mass unit (amu) at 310 °C and 5 µL extracts were directly injected. The compounds were compared with Wiley 275 and the NIST library databases using ChemStation Data Analysis software at 80% quality match.

### Total phenolic content (TPC) and total flavonoid content (TFC)

The Folin–Ciocalteu method (Singleton & Rossi, 1965) was used to determine the TPC of extracts expressed in milligrams of gallic acid equivalents per gram of dry weight (mg GAE/g DW). The aluminum chloride colorimetric method (Tacouri, Ramful-Baboolall & Puchooa, 2013) was used to determine the TFC of the extracts expressed as milligrams of rutin equivalents per gram of dry weight (mg RUE/g DW). Both analyses were determined using a spectrophotometer (Shimadzu, Japan).

### Profiling and quantification of phenolic and flavonoid compounds

The separation of phenolic and flavonoid compounds was performed by high performance–liquid chromatography (HPLC) using an Agilent 1200 series (Agilent Technologies, Santa Clara, CA, USA) equipped with a diode array detector (G1315B 1200) and a 250 mm × 4.6 mm × 5 µm Mightysil RP-18 GP column (Kanto Corp., Portland, Oregon, USA). The mobile phase used was deionized water adjusted to a pH of 2.5 with trifluoroacetic acid (solvent A) and acetonitrile (solvent B). For phenolic profiling, a gradient flow for 70 min was used with a starting ratio of 95:5 (A:B) at an injection volume of 10 µL. For flavonoid profiling, a gradient flow for 33 min was used with a starting ratio of 50:50 (A:B) at an injection volume of 20 µL. Both profiling used a flow rate of 0.6 mL/min and wavelength at 280 nm (phenolic) and 350 nm (flavonoids). Phenolic compounds standards consisted of gallic acid, chlorogenic acid, vanillic acid, caffeic acid, vanillin, 2-hydroxycinnamic acid, syringic acid, p-coumaric acid, epicatechin and flavonoid compounds standards of rutin and quercetin. The quantification was performed with calibration curves using external standards and expressed on a dry weight basis.

### Quantification of capsaicin (C) and dihydrocapsaicin (DHC) contents

The extraction and cleanup procedure using solid phase extraction (SPE) followed the method elaborated by Maokam, Techawongstien & Chanthai (2014). The separation of C and DHC was performed by HPLC using an Agilent 1200 series (Agilent Technologies, Santa Clara, CA, USA) equipped with a diode array detector (G1315B 1200). The column used for the separation was a 250 mm × 4.6 mm × 5 µm Mightysil RP-18 GP column (Kanto Corp, Portland, OR, USA). The mobile phase consisted of 60% acetonitrile and 0.5% formic acid in distilled water with a flow rate of one mL/min and an isocratic flow at ambient temperature for 20 min. The quantification was performed with calibration curves using C and DHC external standards.

### Determination of antioxidant activities

#### 2,2′-azino-bis(3-ethylbenzothiazoline-6-sulfonic acid) (ABTS) scavenging capacity assay

This ABTS^•+^ assay was determined according to the method described by Miller et al. (1993) with a few modifications. Briefly, 7.4 mM ABTS•+ and 2.6 mM potassium persulfate in a ratio of 2:1 (v/v) were mixed to generate the radical cation ABTS. Thereafter, the reaction mixture was incubated in a dark room at room temperature for 14 h and then diluted with methanol to obtain an absorbance of 0.7–0.9 at 734 nm. Subsequently, 100 µL of the extracts were added to 900 µL of diluted ABTS^•+^, homogenized and incubated for 30 min in the dark at room temperature. The absorbance was then measured at 734 nm, and the results were expressed as milligrams Trolox equivalents (TE) per gram of dry weight (mg TE/g DW).

#### 2,2-diphenyl-1-picrylhydrazyl (DPPH) radical scavenging capacity assay

The determination of the DPPH radical-scavenging capacity of the extract was based on the method described by Arnao et al. (1990) with some modifications. Briefly, 0.1 mM DPPH^•^ (900 µL) was added to 100 µL of extracts and incubated for 30 min at room temperature. The absorbance was then measured at 517 nm, and the results were expressed as percentage inhibition. Percentage inhibition was calculated according to the following Eq. (3):

(3) \begin{eqnarray*}\text{Percentage inhibition}(\text{%})= \frac{{\mathrm{A}}_{\mathrm{control}}-{\mathrm{A}}_{\mathrm{sample}}}{{\mathrm{A}}_{\mathrm{control}}} \times 100\end{eqnarray*}

where A_control_ is the absorbance of the control and A_sample_ is the absorbance of the sample.

### Statistical analysis

All tests were conducted in a completely randomized design in independent triplicates to confirm the reproducibility of the results. The report of the data was given as mean ± standard error (SE). Analysis of variance (ANOVA) was performed to assess the bioactive compounds (capsaicinoids, TPC, and TFC) and antioxidant activities. Duncan’s multiple range test determined significant differences, at the 95% confidential level (*p* < 0.05) using statistical SAS software version 9 (SPSS Inc., Chicago, IL, USA).

## Results and Discussion

### Moisture content (MC)

The experimental results were adjusted to estimate the drying conditions (temperatures and times) required to achieve a desired final moisture content. Moisture tests were performed on fresh and dehydrated chili fruits; in all cases, the average initial MC was 75.27% wet basis (w.b.) and the minimum readings were at 12–13% w.b. The weight loss stabilized upon reaching a sample weight ranging 2.4–3.3 g; the final drying time was between 120–1440 min. Figures S1 and S2 show the moisture content as a function of the drying rate; in this case no constant rate period was observed, showing that the weight loss and moisture depended on the drying time for chili fruits for all temperatures. The properties of dried chilli were determined by their MC, since most of its preparation needs the elimination of water. Consequently, the conditions of hot air drying are very important, including control of temperature, velocity, and humidity values, and in many cases, hot air drying is to provide a rapid reduction in the moisture content without affecting the quality.

**Table 1:** Effects of drying temperatures and times on *L* *, *a* *, *b* * values and total color difference (Δ*E*).

Drying time (min)	Moisture content (% w.b.)	L* value	a* value	b* value	Total color difference (Δ*E*)
Fresh	75.27a ± 0.66	40c ± 0.36	45a ± 0.30	27c ± 0.18	–
60 °C					
30	74.78^a^± 1.67	38^d^± 0.68	41^b^± 0.46	24^d^± 0.79	5^g^± 0.47
60	72.13^b^± 0.97	38^d^± 1.08	43^a^± 0.31	24^d^± 0.08	4^g^± 0.23
120	71.98^b^± 0.25	40^c^± 0.23	43^a^± 0.36	27^c^± 0.21	2^h^± 0.15
1440^*^	12.38^k^± 1.49	33^f^± 0.54	26^g^± 1.14	16^e^± 0.34	21^c^± 0.56
80 °C					
30	69.91^bc^± 1.95	36^e^± 0.69	40^b^± 0.18	28^c^± 0.21	5^g^± 0.25
60	69.80^bc^± 2.63	38^d^± 0.34	40^b^± 0.30	26^c^± 0.53	5^g^± 0.12
120	62.49^ef^± 2.60	38^d^± 0.30	41^b^± 0.67	32^b^± 0.21	7^f^± 0.08
600^*^	12.65^k^± 0.18	32^f^± 0.31	27^g^± 0.24	16^e^± 0.67	21^c^± 0.51
100 °C					
30	70.08^bc^± 0.05	38^d^± 0.34	41^b^± 0.61	29^b^± 0.22	5^g^± 0.24
60	66.82^d^± 1.45	39^c^± 0.21	42^a^± 1.64	30^b^± 0.17	5^g^± 0.89
120	60.49^fg^± 0.69	38^d^± 0.70	38^c^± 0.71	28^c^± 0.31	7^f^± 0.31
270^*^	12.28^k^± 0.39	29^g^± 0.65	24^h^± 0.54	13^e^± 0.49	26^b^± 0.29
120 °C					
30	66.67^d^± 0.06	45^a^± 0.19	38^c^± 0.56	32^b^± 0.21	10^e^± 0.08
60	63.76^e^± 0.98	42^b^± 0.69	38^c^± 0.92	28^c^± 0.49	9^e^± 0.19
120	41.03^i^± 1.21	39^c^± 1.23	33^e^± 1.62	26^c^± 1.79	14^d^± 0.65
210^*^	12.91^k^± 0.59	33^f^± 0.59	23^h^± 1.09	16^e^± 1.13	25^b^± 1.42
140 °C					
30	68.42^cd^± 0.76	40^c^± 0.26	36^d^± 0.85	31^b^± 0.89	9^e^± 0.40
60	58.83^g^± 3.78	41^b^± 0.87	38^c^± 0.54	29^b^± 0.81	8^e^± 0.34
120	27.12^j^± 1.36	37^e^± 1.11	29^f^± 1.20	18^e^± 0.89	18^d^± 1.33
180^*^	12.83^k^± 0.90	28^g^± 0.44	18^i^± 0.71	9^f^± 0.47	33^a^± 0.44
160 °C					
30	64.07^e^± 1.05	42^b^± 0.29	38^de^± 0.71	35^a^± 0.57	10^e^± 0.12
60	44.97^h^± 2.62	41^b^± 0.36	38^de^± 0.40	27^c^± 0.48	6^f^± 0.22
120^*^	12.06^k^± 0.08	31^f^± 0.78	25^b^± 1.83	15^e^± 1.34	23^c^± 1.09
180	ND	ND	ND	ND	ND

**Notes.**

Data represents the means ± standard error (*n* = 69) for each sample; mean values with different superscript letters within the same column differ significantly at *p* < 0.05.

* Drying time when the moisture content reached 12–13% w.b.

ND not determined

### Surface color

The measured color values for chilies dried at different temperatures and times are presented in Table 1. At 160 °C, no values were determined or were not determined (ND) at 180 min because the optimum MC (12–13% (wet basis) w.b.) for dried chilies, according to Thai Agricultural Standard (TAS 3001-2010) (Ministry of Agriculture and Cooperatives, 2021), was achieved at 120 min. The *L*^∗^, *a*^∗^ and *b*^∗^ values of fresh chilies were 39.52, 44.51 and 26.54, respectively, with a moisture content of 75.27% w.b. All drying temperatures showed a reduction in the *L*^∗^ value of the chilies when dried to 12–13% w.b. The *a*^∗^ value also showed a significant decrease during drying (*p* < 0.05). The *b*^∗^ value increased during the first 30 min before gradually decreasing with time irrespective of the temperature and was lower in comparison to fresh chilies. The increment during the first 30 min might be due to the decomposition of chlorophyll and carotenoid pigments (Weemaes et al., 1999; Lee & Coates, 1999). On the other hand, the decrease in b* values indicate that the dried chilies were less yellow when dried and are advantageous to produce high-quality paprika (Ergüneş & Tarhan, 2006). The finding was consistent with the results of Minguez-Mosquera & Hornero-Mendez (1994), who reported greater stability of the red pigment compared to the yellow pigment in red pepper. Δ*E*, which compared the color between fresh and dried chilies at different temperatures and times, increased significantly regardless of drying temperatures due to the darkening of the chilies during drying (*p* <0.05). The changes of *L*^∗^*, a*^∗^ and Δ*E* values for drying temperatures 60–100 °C were slower than those at 120–160 °C, which might be due to the rate of non-enzymatic browning (NEB; Maillard reaction and caramelization; Lee et al., 1991) and carotenoid degradation (Yang et al., 2018) that increased with drying temperature and time. According to Lee et al. (1991), the rate of NEB is significantly related to the temperature and water content. However, our study showed that the occurrence of NEB was more dependent on temperature, according to appearance, due to the drying of the whole chili fruits whereby the cells were intact. These intact cells reduced contact between reducing sugar and amino acids that caused NEB. Therefore, high activation energy was required for browning to occur, suggesting a higher temperature dependence on the rate constants. Additionally, significant interaction effects were observed between drying temperature and time for all values in Table 1 (*p* < 0.05). These effects showed that drying temperature influenced the drying speed (drying time), with lower temperature resulting in a longer drying time to achieve the optimum MC. The *L*^∗^, *a*^∗^ and *b*^∗^ values’ decreasing rates were influenced by these interaction effects (*p* < 0.05). The rise in drying temperature has an effect on these values, thus, with an increase in temperature, it was also increased. The same can be seen in the Δ*E* values where a more profound color change was observed with higher drying temperature. These results align with the drying study by Salehi & Kashaninejad (2018) on lemon slices. In their study, the color values were reported to increase with drying time and temperature, associated with nonenzymatic browning such as Maillard reaction and caramelization of sugars.

Daood et al. (2006) reported that carotenoids, primarily capsanthin and capsorubin, which are pigments responsible for the red color in chilies, degraded with higher temperatures (70–100 °C) but were still present in paprika. Moreover, Maillard reactions and other reactions such as pigment degradation that occur during drying cause a dark-brown color of dried chilies, resulting in the decreased a* values (Manzocco et al., 2001). However, the present study showed that chilies subjected to high drying temperatures (120–160 °C) for 210, 180 and 160 min, respectively, remained red in color. This can be explained by Henderson & Henderson (1992) who reported that the stability of carotenoids in the tissue of chilies is affected by the chemical interactions between carotenoids and capsaicinoids, thereby providing substantial protection against thermal degradation. Based on these results, a drying temperature of 60 °C (1440 min) showed the best *L*^∗^, *a*^∗^ and *b*^∗^ values at 12–13% w.b., with the lowest Δ*E* value to maintain the red color of the dried chilies. However, chilies dried at 160 °C up to 12–13% w.b. (120 min) could also maintain the red color based on a* values.

### Bioactive compounds profiling and capsaicinoid derivatives

A total of 32 compounds were identified in both fresh and dried chili samples (Table 2) that can be divided into several classes such as terpenes, pyranone, phenol, lipids, phenolic, alkyl lactone, glycerides, tocopherol, alkaloids, fatty acids and pyrazines. The largest groups of compounds are terpenes (for example, *β*-pinene, limonene and *γ*-terpinene) and fatty acids (for example, myristic acid, undecylic acid and pentadecylic acid). In the terpene group, *β*-pinene and limonene showed a decreasing trend with drying time irrespective of temperature. However, the percentage of overall peak area of limonene at MC of 12–13% w.b. was higher at low temperatures (60–100 °C) compared to high temperatures (120–160 °C). This is because the high temperatures accelerated the chemical reactions (autoxidation) of terpenes, as reported by Lee, Lee & Choe (2007) on different vegetable oils at 25 and 60 °C. Moreover, terpenes are volatile, having a low boiling point and molecular weight, hence exposing the compounds at high temperature with shorter drying time will result in more compound degradation (Hyttinen et al., 2010). However, chilies dried at 160 °C for a short time, 120 min (MC: 12–13% w.b.), still possessed high limonene and pinene. For the fatty acid group, a decrease at the initial drying time before increasing at the end when the MC was 12–13% w.b. was observed. This can be attributed to the reaction between fatty acids and oxygen or the thermal oxidation of polyunsaturated triacylglycerols (Zhang et al., 2012). The MC can affect the oxidation of fatty acids, which is important during storage. Therefore, reducing the MC to 12–13% w.b. can provide dried products with better oxidative stability for long storage periods (Turan, 2018).

**Table 2:** Compounds in ‘Super Hot’ chili fruits dried at different temperatures and times using GC–MS.

**Compounds**	**Percentage of overall peak area (%)**																							
		**60 °C**				**80 °C**				**100 °C**				**120 °C**				**140 °C**				**160 °C**		
	**0**	**30**	**60**	**120**	**1440** ^*^	**30**	**60**	**120**	**600** ^*^	**30**	**60**	**120**	**270** ^*^	**30**	**60**	**120**	**210** ^*^	**30**	**60**	**120**	**180** ^*^	**30**	**60**	**120** ^*^
**Terpenes**																								
*β*-pinene	22^f^± 1	40^c^± 1	38^c^± 1	39^c^± 1	30^e^± 3	45^b^± 4	32^e^± 1	36^d^± 0.1	33^e^± 1	79^a^± 1	38^c^± 1	30^e^± 1	19^g^± 1	40^c^± 2	31^e^± 3	31^e^± 1	33^e^± 1	39^c^± 2	37^d^± 1	30^e^± 0.1	32^e^± 0.1	33^e^± 3	30^e^± 1	25^f^± 1
Limonene	15^f^± 1	35^a^± 1	34^a^± 1	35^a^± 1	24^d^± 1	31^b^± 1	37^a^± 1	34^a^± 0.1	30^b^± 1	12^f^± 0	35^a^± 0.1	30^b^± 1	20^e^± 0.1	35^a^± 1	27^c^± 0.1	27^c^± 0.1	27^c^± 0.1	35^a^± 1	31^b^± 1	25^d^± 0.1	28^c^± 0.1	30^b^± 6	28^c^± 1	23^d^± 0.1
*γ*-terpinene	–	5^a^± 0.1	3^b^± 0.1	3^b^± 0.1	3^b^± 0.1	3^b^± 0.1	3^b^± 0.1	3^b^± 0.1	4^a^± 0.1	1 ± 0.1	4^a^± 0.1	2^b^± 0	3^b^± 0.1	3^b^± 0.1	3^b^± 0.1	3^b^± 0	3^b^± 0.1	3^b^± 0.1	3^b^± 0.1	3^b^± 0.1	3^b^± 0.1	3^b^± 0.1	3^b^± 0.1	3^b^± 0.1
**Pyranone**																								
2,3-dihydro-3,5-dihydroxy-6-methyl-4H-pyran-4-one (DDMP)	13^a^± 1	0.4^c^± 0	1^b^± 0.1	0.5^c^± 0	1^b^± 0.1	0.3^c^± 0.1	0.4^c^± 0.1	0.9^b^± 0.1	0.7^b^± 0	0.3 ± 0.1	1^b^± 0.1	1^b^± 0.1	1^b^± 0.1	1^b^± 0.1	1^b^± 0.1	1^b^± 0	1^b^± 0.1	1^b^± 0.1	1^b^± 0.1	1^b^± 0.1	1^b^± 0.1	0.9^b^± 0.2	0.9^b^± 0.2	0.5^c^± 0.1
**Phenol**																								
2-methoxy-4-vinylphenol	4^a^± 0.1	1^b^± 0.1	0.4^c^± 0.1	1^b^± 0.1	1^b^± 0.1	0.4^c^± 0.1	0.5^c^± 0.1	0.4^c^± 0.1	1^b^± 0.1	0.2^c^± 0.1	0.4^c^± 0.1	1^b^± 0	1^b^± 0.1.1	0.4^c^± 0.1	1^b^± 0.1	1^b^± 0.1	1^b^± 0.1	0.4^c^± 0.1	1^b^± 0	0.8^b^± 0.1	1^b^± 0.1	0.5^c^± 0.1	1^b^± 0	1^b^± 0.1
**Alkaloid**																								
Coniine	–	0.5^b^± 0.1	0.3^b^± 0.1	0.3^b^± 0.1	–	0.2^b^± 0	0.8^a^± 0.2	0.3^b^± 0.1	–	0.1^b^± 0.1	0.7^a^± 0.1	0.4^b^± 0.1	–	0.3^b^± 0.1	–	–	–	0.3^b^± 0.1	0.4^b^± 0.1	–	–	0.4^b^± 0.1	0.4^b^± 0.1	–
Nonivamide	2^a^± 0.1	0.7^b^± 0.1	0.1^c^± 0.1	1^a^± 0.1	2^a^± 0.1	1^a^± 0.1	1^a^± 0.1	1^a^± 0.1	1^a^± 0.1	0.3^c^± 0.1	0.7^b^± 0.1	1^a^± 0.1	3^a^± 0.2	0.8^b^± 0.1	2^a^± 0.1	2^a^± 0.1	2^a^± 0.1	0.7^b^± 0.1	2^a^± 0.1	2^a^± 0.1	2^a^± 0.2	1^a^± 0.1	2^a^± 0.1	2^a^± 0.2
C	8^a^± 0.1	4^c^± 0.1	5^b^± 0.1	5^b^± 0.1	9^a^± 0	5^b^± 0.1	7^b^± 0	6^b^± 0.1	6^b^± 0.1	2^c^± 0.1	4^c^± 0.1	8^a^± 0.1	11^a^± 0.1	4^c^± 0.1	8^a^± 0.1	8^a^± 0	8^a^± 0.1	4^c^± 0.1	8^a^± 0	10^a^± .0.2	11^a^± 0.3	8^a^± 0.3	9^a^± 0.3	12^a^± 1
DHC	6^a^± 0.1	2^c^± 0	3^a^± 0.1	3^b^± 0.1	5^a^± 0.1	3^b^± 0.1	4^b^± 0	4^b^± 0.1	3^b^± .10	1^c^± 0.1	2^c^± 0.1	5^a^± 0.1	7^a^± 0.1	3^b^± 0.1	5^a^± 0.1	5^a^± 0.1	5^a^± 0.1	2^c^± 0.1	5^a^± 0	6^a^± 0.3	6^a^± 0.3	5^a^± 0.1	5^a^± 0.1	7^a^± 0.3
h-C	0.7^a^± 0.1	0.3^c^± 0.1	0.4^b^± 0.1	0.3^c^± 0.1	0.8^a^± 0.1	0.3^c^± 0.1	0.5^b^± 0.1	0.5^b^± 0.1	0.3^c^± 0.1	0.1^c^± 0.1	0.3^c^± 0.1	0.6^b^± 0.1	1^a^± 0.1	0.4^b^± 0.1	0.7^a^± 0.1	0.7^a^± 0.1	0.6^b^± 0.1	0.3^c^± 0.1	0.6^b^± 0.2	0.9^a^± 0.2	1^a^± 0	0.6^b^± 0.2	0.7^a^± 0.1	1^a^± 0.1
h-DHC	–	0.2^c^± 0.1	2^a^± 0	0.2^c^± 0.1	1^a^± 0.1	0.3^c^± 0.1	0.3^c^± 0.1	0.2^c^± 0.1	0.2^c^± 0.1	0.1^c^± 0.1	0.3^c^± 0.1	0.6^b^± 0.1	1^a^± 0.1	0.3^c^± 0.1	0.6^b^± 0.1	0.6^b^± 0.1	–	0.3^c^± 0.1	0.6^b^± 0.2	–	–	0.5^b^± .0.1	–	–
																								
**Phenolic**																								
Vanillin	–	1^a^± 0.1	0.4^b^± 0.1	0.5^b^± 0.1	–	0.4^b^± 0.1	0.5^b^± 0.2	0.5^b^± 0.2	0.7^a^± 0.1	–	0.4^b^± 0.1	0.6^b^± 0.2	0.6^b^± 0.2	0.2^c^± 0.1	0.2^c^± 0.1	0.2^c^± 0.1	0.8^a^± 0.2	0.4^b^± 0.1	0.5^b^± 0.1	0.5^b^± 0.1	1^a^± 0.1	0.5^b^± 0.1	1^a^± 0.1	1^a^± 0.1
**Fatty acid**																								
8-Methyl-6-nonenamide	–	1^a^± 0.1	1^a^± 0.1	1^a^± 0.1	–	–	–	–	–	–	–	–	–	–	–	–	–	–	–	–	–	–	–	–
Myristic acid	3^a^± 0.1	0.4^c^± 0.1	0.3^c^± 0.1	0.2^c^± 0.1	1^a^± 0.1	0.2^c^± 0.1	0.4^c^± 0.1	0.3^c^± 0.1	0.6^b^± 0.1	0.1^c^± 0.1	0.3^c^± 0.1	0.5^b^± 0.1	1^a^± 0.1	0.3^c^± 0.2	0.6^b^± 0.1	0.6^b^± 0.2	0.5^b^± 0.2	0.3^c^± 0.1	0.5b ± 0.2	1^a^± 0.1	0.4^c^± 0.1	0.4^c^± 0.1	0.4^c^± 0.1	1^a^± 0.1
Undecylic acid	2^a^± 0.1	0.7^b^± 0	0.7^b^± 0.2	0.6^b^b ± 0.1	1^a^± 0.1	0.5^b^± 0.1	0.8^b^± 0.2	0.6^b^± 0.2	1^a^± 0.1	0.2^c^± 0.1	1^a^± 0.1	1^a^± 0.2	2^a^± 0.2	0.6^b^± 0.2	1^a^± 0.1	1^a^± 0.1	1^a^± 0.1	1^a^± 0.2	1^a^± 0.1	2^a^± 0.1	1^a^± 0.1	1^a^± 0.1	1^a^± 0.1	2^a^± 0.22
14-Pentadecenoic acid	1^a^± 0.1	0.1^b^± 0	0.1^b^± 0.1	0.1^b^± 0.1	0.3^b^± 0.1	0.1^b^± 0.1	0.1^b^± 0.1	0.1^b^± 0.1	0.2^b^± 0.1	0.1^b^± 0.1	0.1b ± 0.1	0.2^b^± 0.1	0.3^b^± 0.1	0.1^b^± 0.1	0.2^b^± 0.1	0.2^b^± 0.1	0.2^b^± 0.1	0.1^b^± 0.1	0.2^b^± 0.1	0.3^b^± 0.1	0.3^b^± 0.1	0.2^b^± 0.1	0.2^b^± 0.1	0.3^b^± 0.1
Pentadecylic acid	1^a^± 0.1	0.1^b^± 0.0	0.1^b^± 0..0	0.1^b^± 0.1	0.2^b^± 0.1	0.1^b^± 0.1	0.1^b^± 0.1	0.1^b^± 0.1	0.2^b^± 0.1	0.3^b^± 0.1	0.1^b^± 0.1	0.2^b^± 0.1	0.3^b^± 0.1	0.1^b^± 0.1	0.3^b^± 0.1	0.3^b^± 0.1	0.2^b^± 0.1	0.1^b^± 0.1	0.1^b^± 0.1	0.4^b^± 0.1	0.2^b^± 0.1	0.1^b^± 0.1	0.1^b^± 0.1	0.3^b^± 0.1
Palmitoleic acid	2^a^± 0.1	0.7^b^± 0	0.7^b^± 0.2	0.6^b^± 0.1	1^a^± 0.1	0.1^b^± 0.1	1^a^± 0.1	1^a^± 0.1	1^a^± 0.1	0.2^b^± 0.1	0.7^b^± 0.2	1^a^± 0.1	2^a^± 0.2	1^a^± 0.1	1^a^± 0.1	1^a^± 0.1	1^a^± 0.1	1^a^± 0.1	1^a^± 0.1	2^a^± 0.2	1^a^± 0.2	1^a^± 0.1	1^a^± 0.1	2^a^± 0.2
Palmitic acid	3^b^± 0.1	1^b^± 0.3	1^b^± 0.2	1^b^± 0.2	4^a^± 0.2	1^b^± 0.1	2^b^± 0.1	2^b^± 0.2	3^b^± 0.3	1^b^± 0.2	1^b^± 0.2	2^b^± 0.1	5^a^± 0.3	1^b^± 0.1	2^b^± 0.2	2^b^± 0.1	2^b^± 0.2	2^b^± 0.2	2^b^± 0.1	2^b^± 0.2	3^b^± 0.2	2^b^± 0.1	2^b^± 0.1	3^b^± 0.2
Elaidic acid	1^a^± 0.2	0.3^c^± 0.1	0.2^d^± 0.1	0.2^d^± 0.1	0.4^c^± 0.1	0.3^c^± 0.1	0.3^c^± 0.1	0.3^c^± 0.1	0.5^b^± 0.1	0.1^d^± 0.1	0.3^c^± 0.1	0.3^c^± 0.1	0.5^b^± 0.1	0.3^c^± 0.1	0.4^c^± 0.2	0.4^c^± 0.1	0.3^c^± 0.2	0.4^c^± 0.1	0.4^c^± 0.1	0.6^b^± 0.1	0.4^c^± 0.1	0.5^b^± 0.1	0.5^b^± 0.1	0.4^c^± 0.1
Margaric acid	1^a^± 0.1	0.1^c^± 0	0.1^c^± 0.1	0.1^c^± 0.1	0.3^b^± 0.1	0.1^c^± 0.1	0.1^c^± 0.0	0.1^c^± 0.1	0.2^c^± 0.1	–	0.2^c^± 0.1	–	0.4^b^± 0.1	–	0.3^b^± 0.1	0.3^b^± 0.1	0.3^b^± 0.1	–	0.2^c^± 0.1	0.4^b^± 0.1	0.3^b^± 0.1	–	–	–
Oleic acid	1^a^± 0.1	0.1^c^± 0.0	0.1^c^± 0.1	0.1^c^± 0.1	0.3^b^± 0.1	0.1^c^± 0.0	0.1^c^± 0.0	0.1^c^± 0.0	0.2^c^± 0.1	0.03^d^± 0.0	0.1^c^± 0.0	0.2^c^± 0.1	0.3^b^± 0.1	0.1^c^± 0.1	0.2^c^± 0.1	0.2^c^± 0.1	0.2^c^± 0.1	0.2^c^± 0.1	0.2^c^± 0.1	0.3^b^± 0.1	0.3^b^± 0.1	0.2^c^± 0.1	0.2^c^± 0.1	0.3^b^± 0.1
Methyl vaccenate	1^a^± 0.1	0.3c ± 0.1	0.2c ± 0.1	0.3c ± 0.1	0.4^b^± 0.1	0.3c ± 0.1	0.3c ± 0.1	0.3c ± 0.0	0.5^b^± 0.1	0.1^c^± 0.1	0.3^c^± 0.1	0.6^b^± 0.1	0.3^c^± 0.1	0.3^c^± 0.1	1^a^± 0.1	1^a^± 0.1	1^a^± 0.1	0.4^b^± 0.1	1^a^± 0.1	0.3^c^± 0.1	1^a^± 0.1	0.4^b^± 0.1	0.5^b^± 0.2	0.5^b^± 0.2
*α*-Linolenic acid	3^b^± 0.2	2^c^± 0.2	2^c^± 0.2	2^c^± 0.2	6^a^± 0.2	2^c^± 0.1	3^b^± 0.1	3^b^± 0.1	5^a^± 0.2	0.8^d^± 0.1	2^c^± 0.1	4^b^± 0.2	6^a^± 0.2	2^c^± 0.1	4^b^± 0.1	4^b^± 0.1	4^b^± 0.1	3^b^± 0.1	3^b^± 0.1	4^b^± 0.2	4^b^± 0.2	2^c^± 0.1	3^b^± 0.1	4^b^± 0.2
Stearic acid	2^b^± 0.3	1^b^± 0.2	1^b^± 0.1	1^b^± 0.1	2^b^± 0.2	1^b^± 0.1	1^b^± 0.1	1^b^± 0.1	2^b^± 0.1	0.5^c^± 0.1	1^b^± 0.1	2^b^± 0.1	4^a^± 0.2	1^b^± 0.1	–	–	–	2^b^± 0.1	–	–	2^b^± 0.1	2^b^± 0.2	2^b^± 0.1	3^a^± 0.1
																								
**Lipids**																								
2-Palmitoylglycerol	1^a^± 0.1	1^a^± 0.2	1^a^± 0.1	1^a^± 0.2	1^a^± 0.1	1^a^± 0.2	1^a^± 0.1	1^a^± 0.1	1^a^± 0.1	0.5^b^± 0.1	1^a^± 0.1	2^a^± 0.1	1^a^± 0.2	1^a^± 0.1	2^a^± 0.2	2^a^± 0.2	2^a^± 0.2	1^a^± 0.1	1^a^± 0.2	–	2^a^± 0.3	2^a^± 0.3	2^a^± 0.2	1^a^± 0.1
(-)- *β*-Elemene	–	0.03^b^± 0.1	0.05^b^± 0.0	0.06^b^± 0.0	0.2^a^± 0.1	0.04^b^± 0.0	0.06^b^± 0.0	0.06^b^± 0.0	0.03^b^± 0.0	–	0.03^b^± 0.0	0.07^b^± 0.0	0.2^a^± 0.1	0.04^b^± 0.0	0.1^a^± 0.0	0.1^a^± 0.0	0.1^a^± 0.1	0.03^b^± 0.0	0.1^a^± 0.2	0.1^a^± 0.1	0.2^a^± 0.1	0.1^a^± 0.1	0.1^a^± 0.0	0.1^a^± 0.0
Nerolidyl acetate	0.2^c^± 0.1	0.1^c^± 0.1	0.1^c^± 0.1	0.2^c^± 0.1	0.3^c^± 0.1	0.4^b^± 0.1	0.4^b^± 0.1	0.5^b^± 0.0	0.5^b^± 0.1	0.2^c^± 0.1	0.4^b^± 0.0	1^a^± 0.1	–	0.5^b^± 0.0	–	–	–	0.5^b^± 0.1	0.5^b^± 0.2	–	0.4^b^± 0.1	1^a^± 0.1	1^a^± 0.2	–
**Alkyl lactone**																								
Massoia lactone	1^a^± 0.1	0.3^b^± 0.1	0.4^b^± 0.1	0.4^b^± 0.1	1^a^± 0.1	1^a^± 0.1	0.4^b^± 0.1	0.4^b^± 0.1	–	0.1^b^± 0.0	0.2^b^± 0.0	0.5^b^± 0.1	1^a^± 0.3	0.3^b^± 0.1	0.4^b^± 0.0	0.4^b^± 0.0	0.5^b^± 0.1	0.3^b^± 0.2	0.4^b^± 0.1	1^a^± 0.2	1^a^± 0.2	0.4^b^± 0.2	0.5^b^± 0.2	1^a^± 0.3
**Pyrazine**																								
Isopropyl methoxy pyrazine	0.3^a^± 0.1	0.1^a^± 0.1	0.1^a^± 0.1	0.1^a^± 0.0	0.3^a^± 0.1	0.2^a^± 0.1	0.2^a^± 0.0	0.2^a^± 0.1	0.1^a^± 0.0	0.1^a^± 0.0	0.1^a^± 0.0	0.2^a^± 0.1	0.3^a^± 0.1	0.1^a^± 0.0	0.2^a^± 0.0	0.2^a^± 0.1	0.2^a^± 0.1	0.1^a^± 0.0	0.2^a^± 0.1	0.3^a^± 0.1	0.3^a^± 0.2	0.2^a^± 0.1	0.2^a^± 0.1	0.3^a^± 0.1
**Glycerides**																								
Stearic acid *α*-monoglyceride	1^a^± 0.1	0.2^b^± 0.1	0.3^b^± 0.1	0.2^b^± 0.1	0.4^b^± 0.1	0.1^b^± 0.0	0.4^b^± 0.1	0.3^b^± 0.1	2^a^± 0.3	0.3b ± 0.0	0.4b ± 0.1	1^a^± 0.2	1^a^± 0.1	0.3^b^± 0.1	0.6^b^± 0.1	0.6^b^± 0.2	0.6^b^± 0.2	0.4^b^± 0.2	1^a^± 0.2	0.5^b^± 0.1	1^a^± 0.2	1^a^± 0.2	0.4^b^± 0.1	1^a^± 0.2
**Other**																								
Vitamin E	2^a^± 0.1	0.4 ± 0.1	1^a^± 0.1	0.4 ± 0.1	1^a^± 0.1	0.5^b^± 0.1	0.5^b^± 0.0	0.5^b^± 0.1	1^a^± 0.2	0.1^b^± 0.0	1^a^± 0.1	1^a^± 0.1	2^a^± 0.2	0.6^a^± 0.2	1^a^± 0.3	1^a^± 0.3	2^a^± 0.2	1^a^± 0.3	1^a^± 0.2	2^a^± 0.2	2^a^± 0.1	1^a^± 0.2	1^a^± 0.1	0.4^a^± 0.1

**Notes.**

Data represents the means ± standard error (*n* = 69) for each sample.

* Drying time when moisture content reached 12–13% w.b.; ‘–’ = not detected.

C capsaicin DHC dihydrocapsaicin h-C homocapsaicin h-DHC homodihydrocapsaicin

The same trend is also evident in the alkaloid group. Capsaicinoids were higher in dried chilies than in fresh chilies and increased at high temperatures (120–160 °C), consequently increasing the pungency. Interestingly, the compound coniine was also detected at high temperatures (120–160 °C) between 30 and 60 min. This compound, found in dried chilies, can be a potential source of antioxidants (Irabor et al., 2021). The pyrazine group compound was detected in dried chilies irrespective of temperature and time. The formation of this pyrazine group was due to the Maillard reaction that facilitated the condensation between amino compounds and sugar fragments (Yu et al., 2013) during the drying process. Furthermore, vitamin E also increased in chili samples except at 160 °C. This can be attributed to an increased extractability due to the release of their components to their binding sites during heating (Hwang, Stacewicz-Sapuntzakis & Bowen, 2012). A small percentage of the overall peak area of vanillin was also detected; however, the percentages of overall peak area did not differ among temperatures at 12–13% w.b. (*p* < 0.05). The presence of vanillin could be due to thermal cleavage of capsaicinoids between an amide bond and vanillin moiety (Henderson & Henderson, 1992) or from the oxidation of ferulic acid (Kundu, 2017). These results indicate that bioactive compounds were still detected at a high drying temperature of 160 °C in dried chilies and possessed nutritive values.

**Table 3:** Total phenolic and flavonoid contents as affected by different drying temperatures and times.

Drying time (min)	Total phenolic content (mg GAE/g DW^ψ^)	Total flavonoid content (mg RUE/g DW^ψ^)	Capsaicin (mg C/g DW)	Dihydrocapsaicin (mg DHC/g DW)
0	36.78^hij^± 1.15	257.35^a^± 3.30	0.39^d^± 0.01	0.28^k^± 0.00
60 °C				
30	42.19^dfh^± 3.71	131.41^ghi^± 3.77	2.63^bc^± 0.23	1.51^cfg^± 0.14
60	54.65^b^± 0.98	115.26^ijk^± 7.27	3.64^a^± 0.28	1.83^ab^± 0.11
120	42.93^df^± 0.72	161.12^cd^± 1.48	2.83^abc^± 0.34	1.54^bcf^± 0.11
1440^*^	36.68^hij^± 1.01	135.69^gh^± 1.72	2.25^c^± 0.40	1.51^cfg^± 0.10
80 °C				
30	56.36^b^± 2.04	76.72m ± 13.11	2.32^c^± 0.31	1.21^gh^± 0.06
60	39.46^fi^± 2.48	98.99^kl^± 5.89	3.24^ab^± 0.36	2.03^a^± 0.16
120	32.45^j^± 2.49	87.69^lm^± 5.40	2.02^c^± 0.23	1.31^efgh^± 0.09
600^*^	38.00^ghij^± 0.73	119.50^hij^± 4.07	1.01^d^± 0.55	1.31^efgh^± 0.09
100 °C				
30	43.34^ef^± 0.77	145.11^defg^± 7.03	2.53^bc^± 0.25	1.62^bc^± 0.06
60	42.18^efh^± 0.67	107.45^jk^± 5.17	2.27^c^± 0.05	1.33^dfgh^± 0.01
120	47.45^cd^± 1.45	159.46^cd^± 6.32	2.15^c^± 0.32	1.46^cfg^± 0.09
270^*^	40.09^fhi^± 3.52	154.02^def^± 4.49	2.25^c^± 0.31	1.25^fgh^± 0.04
120 °C				
30	63.54^a^± 0.29	176.40^bc^± 3.74	2.23^c^± 0.15	1.40^cfg^± 0.08
60	67.15^a^± 1.82	139.52^efg^± 2.54	2.25^c^± 0.33	1.26^fgh^± 0.19
120	53.40^b^± 2.83	142.93^defg^± 6.38	2.02^c^± 0.40	1.61^bce^± 0.01
210^*^	39.95^hi^± 3.28	154.00^def^± 3.01	2.28^c^± 0.18	1.37^cfg^± 0.13
140 °C				
30	39.59^fhi^± 2.56	179.40^b^± 5.81	0.83^d^± 0.37	0.49^jk^± 0.21
60	41.43^fh^± 1.66	120.66^hij^± 2.60	2.82^abc^± 0.49	1.40^cfg^± 0.14
120	51.55^bc^± 1.36	157.25^de^± 12.27	2.02^c^± 0.40	1.04^hi^± 0.06
180^*^	51.03^bcd^± 4.63	177.39^bc^± 16.94	2.28^c^± 0.54	0.74^ij^± 0.13
160 °C				
30	45.27^df^± 0.48	137.09^fgh^± 4.99	3.27^ab^± 0.06	1.98^a^± 0.06
60	35.50^ij^± 0.85	160.81^cd^± 2.60	2.52^bc^± 0.29	1.65^bc^± 0.03
120^*^	55.42^b^± 3.21	228.40^a^± 4.52	2.52^bc^± 0.16	1.58^bce^± 0.07
180	ND	ND	ND	ND

**Notes.**

Data represents the means ± standard error (*n* = 69) for each sample; mean values with different superscript letters within the same column differ significantly at *p* < 0.05

* Drying time when moisture content reached 12–13% w.b.

^ψ^ at moisture content of each sampling time

ND not determined

### Total phenolic content (TPC)

The TPC in chili extracts was ascertained for different drying temperatures and times (Table 3) with fresh chilies with a value of 36.78 mg GAE/g DW. TPC values at 120 °C for 30 min (63.54 mg GAE/g DW) and 60 min (67.15 mg GAE/g DW) were significantly the highest among the drying temperatures (*p* < 0.05). At low temperatures (60–00 °C), the TPC gradually decreased with drying time. However, at 12–13% w.b., the TPC was not different from that of fresh chilies. Moreover, TPC values for drying temperatures of 140 °C and 160 °C at 180 min and 120 min, respectively, were higher than those of fresh chilies by 27.9% and 33.6%, respectively. Therefore, the overall trend of TPC during the drying time showed an initial increase, followed by a decrease and finally an increase. Drying temperatures and time had significant interaction effect on TPC ( *p* < 0.05). These effects concur with studies by Multari et al. (2018), who suggested that higher drying temperatures can enhance the phenolic compounds’ solubility, leading to the collapse of cellular structures. Consequently, the release of phenolic compounds that are bound to the macromolecules of the cell wall is enhanced. During drying, several chemical processes occur concurrently that can either reduce or increase the TPC. The TPC of chilies dried at low temperatures (60–100 °C) will result in oxidation due to the increased contact between oxidative enzymes and their substrates (phenolic compounds) as the membrane loses its selective permeability (Torki-Harchegani et al., 2016). Conversely, chilies dried at high temperatures (140 °C and 160 °C) showed a potential increase in TPC as the compounds disassociate from the proteins or the cell wall due to their hydrophobic benzenoid rings and hydrogen-bonding potential of the phenolic hydroxyl groups, which are heat-labile (Asano et al., 1982). However, heat treatments caused the cleavage of glycoside- and ester-bound fractions of phenolic acids, subsequently reducing the TPC (Hayat et al., 2010). The same result was also observed by Zhou et al. (2016), who reported an increase in TPC degradation with temperatures. This is in contrast with the current study that showed higher TPC values in chilies dried at 140 °C (51.03 mg GAE/g DW) and 160 °C (55.42 mg GAE/g DW) for 180 min and 120 min, respectively, compared to fresh chilies. Besides the compounds being released into the free state, the increment in TPC could also be attributed to the by-products and intermediates from the Maillard reaction described in Table 2. Although drying at 120 °C for 30 min and 60 min had the highest TPC, the shelf life of dried chilies was reduced due to the high MC content (>60% w.b.). However, drying with 160 °C for 120 min (12.06% w.b.) could increase the shelf life but with a slight reduction in TPC.

**Table 4:** Phenolic contents (gallic acid, chlorogenic acid, vanillic acid, caffeic acid, syringic acid, vanillin, *p*-coumaric and 2-hydroxycinnamic acid) and flavonoid contents (rutin and quercetin) as affected by different drying temperatures and times.

Drying time (mins)	mg/g dry weight^ψ^									
	Gallic acid	Chlorogenic acid	Vanillic acid	Caffeic acid	Syringic acid	Vanillin	*p*-coumaric	2-hydroxycinnamic acid	Rutin	Quercetin
Fresh	–	8.24^hi^± 0.08	–	–	0.36^ab^± 0.06	–	–	–	4.24^b^± 0.04	0.03^bc^± 0.01
60 °C	–		–	–						
30	–	0.75^j^± 0.07	–	–	0.35^b^± 0.07	–	–	–	3.13^e^± 0.07	–
60	–	8.73^h^± 0.05	–	1.14^d^± 0.05	–	–	–	–	3.41^d^± 0.12	–
120	–	7.41^i^± 0.05	–	1.36^c^± 0.06	–	–	–	–	2.54^gh^± 0.08	–
1440^*^	–	1.53^j^± 0.03	0.45^f^± 0.11	–	–	–	–	–	1.62^k^± 0.02	0.02^bc^± 0.01
80 °C	–									
30	–	14.33^c^± 0.68	0.65^e^± 0.06	1.54^a^± 0.04	–	–	–	–	3.25^de^± 0.13	–
60	–	12.05^d^± 0.22	0.63^e^± 0.04	1.37^c^± 0.05	–	–	–	–	2.64^fg^± 0.07	–
120	–	17.25^b^± 0.14	1.06^c^± 0.06	1.46^b^± 0.03	0.43^a^± 0.04	–	–	–	3.85^c^± 0.06	–
600^*^	0.48^d^± 0.05	16.69^b^± 0.39	1.87^a^± 0.05	0.85^e^± 0.03	0.18^c^± 0.03	–	–	–	2.83^f^± 0.05	0.02^bc^± 0.01
100 °C										
30	–	11.82^de^± 0.25	–	–	–	–	–	–	2.73^fg^± 0.07	–
60	–	8.66^h^± 0.38	–	–	–	–	–	–	1.76^k^± 0.06	–
120	–	14.31^c^± 0.23	0.78^d^± 0.05	–	–	–	–	–	3.48^d^± 0.04	0.03^ab^± 0.00
270^*^	0.41^e^± 0.05	12.57^d^± 0.54	1.22^b^± 0.05	–	–	–	–	0.05^b^± 0.00	2.58^gh^± 0.16	0.03^ab^± 0.00
120 °C										
30	–	14.75^c^± 0.26	0.06^g^± 0.01	–	–	–	–	–	4.29^b^± 0.06	0.04^a^± 0.01
60	–	20.61^a^± 0.32	0.96^c^± 0.05	–	–	–	–	0.07^a^± 0.01	5.15^a^± 0.07	0.03^ab^± 0.00
120	–	11.31^def^± 0.55	0.62^e^± 0.01	–	–	–	–	–	2.56^gh^± 0.05	0.02^bc^± 0.00
210^*^	0.47^de^± 0.05	12.37^d^± 0.49	1.15^b^± 0.06	–	–	–	–	0.04^c^± 0.01	2.37^hi^± 0.07	0.02^bc^± 0.00
140 °C										
30	–	12.24^d^± 0.48	–	–	–	–	–	–	3.29^de^± 0.04	–
60	–	10.60^efg^± 0.49	–	–	–	–	–	–	2.75fg ± 0.12	–
120	1.29^c^± 0.04	11.41^def^± 0.49	–	–	–	–	–	0.04^c^± 0.01	2.78^fg^± 0.07	0.02^bc^± 0.00
180^*^	5.94^b^± 0.05	15.42^c^± 0.50	–	–	–	–	–	0.05^b^± 0.00	2.77f^g^± 0.03	0.02^bc^± 0.00
160 °C			–	–	–	–	–			
30	–	8.58^hi^± 0.47	–	–	–	–	–	–	2.55^gh^± 0.05	–
60	0.21^f^± 0.02	9.46^gh^± 0.61	–	–	–	–	–	–	2.26^ij^± 0.07	–
120^*^	6.83^a^± 0.06	10.47^fg^± 0.51	–	0.44^f^± 0.04	0.37^ab^± 0.06	–	–	–	2.11^j^± 0.07	0.01^d^± 0.00
180	ND	ND	ND	ND	ND	ND	ND	ND	ND	NA

**Notes.**

Data represents the means ± standard error (*n* = 69) for each sample; mean values with different superscript letters within the same column differ significantly at *p* < 0.05.

* Drying time when moisture content reached 12–13% w.b.

^ψ^ at moisture content of each sampling time.

ND not determined – not detected

### Profiling and quantification of phenolic compounds

A total of nine phenolics (gallic acid, chlorogenic acid, vanillic acid, caffeic acid, syringic acid, vanillin, p-coumaric acid, epicatechin and 2-hydroxycinnamic acid) alongside 18 unknown compounds were detected in the chili extracts by HPLC (Table 4). The overall trend observed was a higher increment in phenolic compounds at drying temperatures of 120–160 °C versus 60–100 °C at 12–13% w.b. by 1.2 times and with drying time. Chlorogenic acid was the most stable and abundant compound amongst all the quantified phenolic compounds (0.77–20.83 mg/g DW). However, vanillin and p-coumaric acid were not detected in all of the chili extracts irrespective of temperature and time. This might be caused by the degradation of vanillin to 2-methoxy-4-vinylphenol (Henderson & Henderson, 1992) or it being used for C biosynthesis. However, p-coumaric acid can be degraded to 4-vinylphenol (Barthelmebs, Divies & Cavin, 2000) and chlorogenic acid (Gramazio et al., 2014). Consequently, chlorogenic acid increased in all drying temperatures except at 60 °C. Vanillic acid and caffeic acid were also detected, consistent with the previous study (Shaimaa et al., 2016). Vanillic acid was only detected at the temperatures of 60 °C (0.44–1.72 mg/g DW), 80 °C (0.62–1.88 mg/g DW), 100 °C (0.78–1.22 mg/g DW) and 120 °C (0.05–1.18 mg/g DW), while caffeic acid was not detected at drying temperatures of greater than 80 °C as vanillic acid is less susceptible to oxidation than caffeic acid (Réblová, 2012). Furthermore, vanillic acid can also be formed from the oxidation of vanillin (Kundu, 2017). The oxidation product of a phenolic compound such as caffeic acid that yields dehydrodicaffeic acid dilactone can also influence the TPC pool (Antolovich et al., 2004). Moreover, these compounds can also be reduced by binding with other compounds, oxidation and irreversible structural changes as polyphenolics are heat-labile (Réblová, 2012).

### Total flavonoid content (TFC)

The TFC irrespective of drying time and temperature was less than that of fresh chilies (257.35 mg RUE/g DW; Table 3) but TFC were in the order of 160 °C >140 °C >120 °C = 100 °C >80 °C <60 °C at 12–13% w.b. (*p* < 0.05). The TFC tended to decrease during the first 60 min before increasing at the end (12–13% w.b.), yet the levels remained lower than that in fresh chilies. These results were due to flavonoid degradation through oxidation (Buchner et al., 2006) when the cells rupture from heat treatment (Torki-Harchegani et al., 2016). At 12–13% w.b., high temperatures (120–160 °C) had higher TFC values than low temperatures (60–100 °C). This can be due to the faster removal of MC from the chilies that causes the deactivation of degradative enzymes (Korbel et al., 2013). Furthermore, the increment in TFC may be due to the release of flavonoids from the cell wall by disrupting hydrogen bonds (Asano et al., 1982), hydroxylation that yielded additional compounds such as protocatechuic acid and glycosylation of flavonoids such as rutin (Chaaban et al., 2016). In terms of interaction, TFC showed the same trend as TPC, whereby the contents increased with temperature and time. This result implied that drying at high temperatures such as 160 °C can sustain TFC values in ‘Super Hot’ chilies.

### Profiling and quantification of flavonoid compounds

Rutin and quercetin are the primary flavonoids in chilies and were quantified in this study. The results showed that in fresh chilies, both rutin (4.22 mg/g DW) and quercetin (0.03 mg/g DW) were detected alongside 15 unknown compounds (Table 4). Rutin (1.62–5.17 mg/g DW) was observed to be more thermostable compared to quercetin and was detected in all chili extracts, irrespective of time and temperature. Interestingly, rutin and quercetin were detected at a high temperature of 160 °C. However, rutin showed a decreasing trend with drying time and was 2–3 times lower than fresh chilies. For quercetin, only a small concentration can be detected in fresh samples (0.03 mg/g DW) and dried samples (0.01–0.08 mg/g DW) with higher degradation at high temperatures (120–160 °C) than at low temperatures (60–100 °C). The reduction in the rutin and quercetin contents might be because of the transfer of hydrogen atoms from flavonols, hydroxylation and glycosylation (Chaaban et al., 2016; Buchner et al., 2006). The degradation rates from drying between flavonoids also differ depending on the structure such as different numbers and arrangements in the hydroxyl groups (Rice-Evans, Miller & Paganga, 1996). Furthermore, the integrity of the cell structure will be compromised during drying, resulting in migration of the compounds and consequently the breakdown or degradation of the compounds due to various chemical reactions from degradative enzymes, light and oxygen (Torki-Harchegani et al., 2016). Temperatures of 60–100 °C caused the slow removal of MC within the chili cells, thus a higher degradative enzyme activity compared to temperatures of 120–160 °C (Korbel et al., 2013).

### Quantification of capsaicin (C) and dihydrocapsaicin (DHC) contents

Both C and DHC are the most abundant capsaicinoids in chili fruits (Maokam, Techawongstien & Chanthai, 2014) that contribute the most to the pungency and spiciness in hot chilies. Therefore, only C and DHC were quantified in the chili samples (Table 3; Figs. S3 and S4). In fresh chilies, the C content was 0.39 mg C/g DW whereas the DHC content was 0.28 mg DHC/g DW. The highest C contents could be found at drying temperatures 60 °C for 60 min, 80 °C for 60 min, 140 °C for 30 min and 160 °C for 30 min. Whereas, drying at 80 °C for 60 min and 160 °C for 30 min resulted in the highest DHC content (*p* < 0.05). Drying temperature and time significantly influenced the C and DHC contents, showing low contents. According to Martín-Cabrejas et al. (2009), thermal degradation and the release of capsaicinoids may occur concurrently, but the contents depends on the maximum degradation occuring at that temperature. The C and DHC contents were also higher than that of fresh chilies irrespective of temperature and time with an average of a 5-fold increase. The increase in capsaicinoid contents was in line with the study by Popelka et al. (2017), who found that drying chilies to approximately 10–11% w.b. MC resulted in the increasing C contents 71–92%, depending on the variety. Contradictorily, Zhou et al. (2016) reported a decrease of 18% in the contents during drying. These studies had a capsaicin content of 1.13–25.94 mg C/g DW (depending on chili variety) and 0.52–0.56 mg C/g DW. Comparatively, the current results had a higher C contents than the aforementioned studies. The results also showed that C and DHC started being released from the cell matrix within 30 min of drying due to their dissociation from the cell structures. Overall, at the end drying times at 12–13% MC (1440, 600, 270, 210, 180 and 120 min), the C contents increased by an average of six times compared to fresh chilies, with the greatest increase at 140 °C (seven times) and the smallest increase at 80 °C (three times). Oxidation of C and DHC occurred during drying when cells ruptured and with longer time needed to remove moisture. This facilitated the oxidation reaction between C, DHC and degradative peroxidase, which are in proximity. Conversely, at high temperatures (120–160 °C), the rapid removal of moisture from the chili fruits can influence the peroxidase activity and consequently the C and DHC contents as well. In the present study, the shorter time needed to achieve 12–13% w.b. at high temperatures (120–160 °C) was beneficial in improving the C and DHC contents that might be contributed by the reduction in peroxidase activity. This is in agreement with the study by Korbel et al. (2013) who observed that the peroxidase activity was reduced at a temperature of 60 °C compared to 50 °C with low water content. The availability of C and DHC was also higher at 12–13% w.b. than fresh chilies because both C and DHC were released from the bound state into the free state (Asano et al., 1982) and also due to their hydrophobicity and high boiling point. The thermal decomposition of C and DHC occurred at high temperature (160 °C) that may be caused by the cleavage between an amide bond and vanillin moiety, hydrogenation or the arrangement of methylnonenamide and deamination of 8-methyl-6-nonemide (Henderson & Henderson, 1992); however, the contents were higher and stable throughout the drying period. Additionally, the degradation of capsaicinoids is also attributed to chemical changes in the structure, such as deprotonation of the hydroxyl group (Suresh, Manjunatha & Srinivasan, 2007) or oxidation reactions catalyzed by peroxidase at low drying temperatures. This result implies that the whole chili fruits dried at 160 °C for 120 min induced the bioactive compounds through their disassociation from the cell wall (placenta) and quicker removal of water that increased the concentration.

### Antioxidant capacities

**Figure 1:**
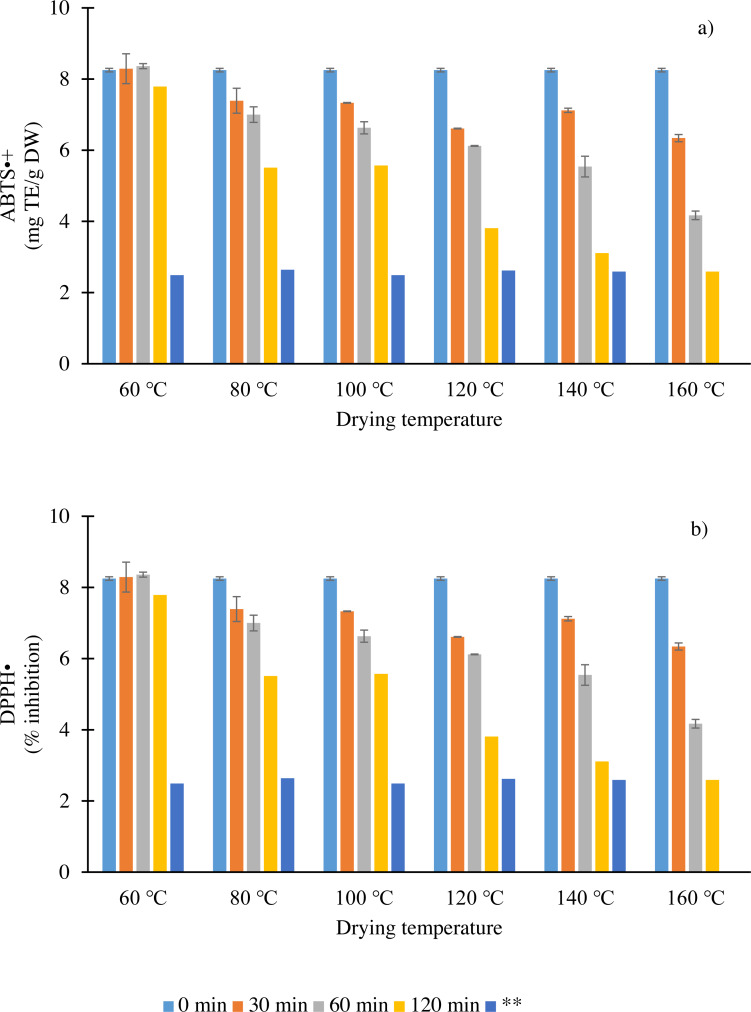
Antioxidant capacity (ABTS^•+^ (A) and DPPH^•^ (B) assays) as affected by different drying temperatures and times. Vertical bars represent standard error for each sample; asterisks (**) indicate drying time when moisture content reached 12–13% w.b.

Dried chilies are consumed not only for their high polyphenol contents and capsaicinoids but also for their antioxidant capacities. Therefore, the antioxidant capacities of dried chilies at different temperatures and times were evaluated in terms of ABTS and DPPH radical scavenging activity assays (Fig. 1). The antioxidant capacities in fresh chilies were 8.25 mg TE/g DW (ABTS^•+^ assay) and 90.81% inhibition (DPPH^•^ assay). An interaction effect was seen in the antioxidant activities of dried chilies, indicating that the compounds have lost a few of their scavenging capacities. Moreover, this decline may be partially related to the reduction, oxidative degradation and polymerization–condensation of some compounds (C, DHC, rutin and vanillic acid) (Pedroza et al., 2012; Teles et al., 2018). In ABTS^•+^ and DPPH^•^ assays, a reaction with a hydrogen donor renders the solution colorless (Pisoschi & Negulescu, 2011). Both ABTS^•+^ and DPPH^•^ decreased with time irrespective of drying temperature, with average losses of 0.6 and 0.9 times, respectively. In addition, the results showed that the decrease in antioxidant capacities was also temperature-dependent. The reduction in both assays showed that the ability of the antioxidants, such as capsaicinoids, vanillic acid and rutin, to donate hydrogen was weakened presumably due to the cleavage of hydroxyl groups from the structure by heat (Chaaban et al., 2016), although the content increased. However, Chaaban et al. (2016) reported that the antioxidant capacity has either a higher, lower or same activity as the native compounds, depending on the number of hydroxyl groups in their structures, due to the change in or degradation of polyphenol structures, such as glycosylation and alkylation, which gives rise to new products. Based on these results, a stable antioxidant capacity was observed when chilies were dried at 160 °C for a shorter time (120 min) compared to other temperatures.

## Conclusions

The results of this study confirm the hypothesis that drying temperatures and time influences the capsaicinoids content, phenolic and flavonoid contents and antioxidant capacity in ‘Super Hot’ chilies. In the present study, the dried samples can be classified into two groups: (a) low drying temperatures (60–100 °C) that slightly affects the TPC, C, DHC and antioxidant activities of dried chilies and (b) high drying temperatures (120–160 °C) that caused physiochemical changes in color and compound contents. When the MC dropped below 50%, the TFC and surface color (*a*^∗^*, b*^∗^ and Δ*E*) of dried chilies were affected. Although the main compounds present in chili fruits were capsaicinoids, TPC, TFC, limonene, pinene, tocopherol and oleic acid, regardless of drying temperature and time, the bioactive compounds (C, DHC, TPC and TFC) were induced at drying temperature of 160 °C. At the end of drying time, MC was 12–13% w.b., TFC decreased, TPC and the main pungent compounds, capsaicin and dihydrocapsaicin increased, but these compounds were higher than the fresh chilies. However, the antioxidant capasities (ABTS^•+^ and DPPH^•^) of dried chilies at all temperatures decreased with increasing drying time and less than fresh chilies. This is the first report that reveals a higher pungency in dark red dried chilies as compared to lighter red dried chilies, as indicated by the capsaicinoid contents. Thus, hot air drying at a high temperature (160 °C for 120 min) as an alternative and inexpensive postharvest treatment is a new finding to maintain or improve the bioactive compounds in dried chilies, especially capsaicinoids contents and to reduce the production time compared to low drying temperature condition (60 °C for 1440 min).

## Supplemental Information

10.7717/peerj.13423/supp-1 Table S1 Raw data for surface color of chilies (L*, a*, b* and color difference)

10.7717/peerj.13423/supp-2 Table S2 Raw data for Table 2

10.7717/peerj.13423/supp-3 Table S3 Raw data for total phenolic and flavonoid content

10.7717/peerj.13423/supp-4 Table S4 Raw data for phenolic compounds

10.7717/peerj.13423/supp-5 Supplemental Information 5 Raw data for flavonoid contents

10.7717/peerj.13423/supp-6 Supplemental Information 6 Raw data for capsaicin and dihydrocapsaicin

10.7717/peerj.13423/supp-7 Figure S1 Raw data for antioxidant capacity

10.7717/peerj.13423/supp-8 Supplemental Information 8 Supplementary Figures

## Additional Information and Declarations

### Data Availability

The following information was supplied regarding data availability: The raw measurements used for statistical analysis to compare the treatments are available in the Supplemental Files.
