# Supplementary material for: Influence of hot air drying on capsaicinoids, phenolics, flavonoids and antioxidant activities of ‘Super Hot’ chilies
Source: PeerJ. 2022 May 25;10:e13423. doi: 10.7717/peerj.13423 (PMC9147319; doi:10.7717/peerj.13423)
Supplement: Supplemental Information 8 [file peerj-10-13423-s008.docx]

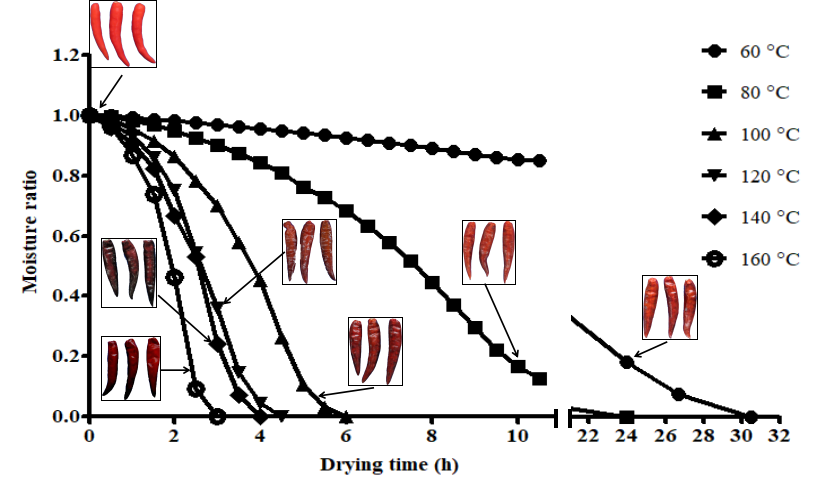


**Figure S1** Drying curves for chili at different drying temperatures.


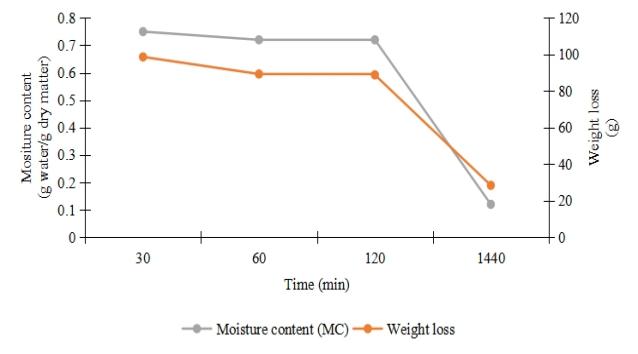

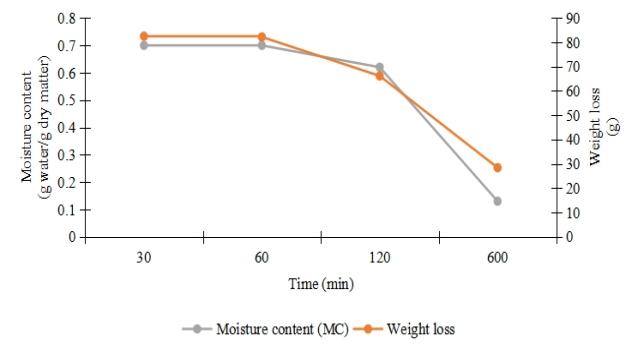

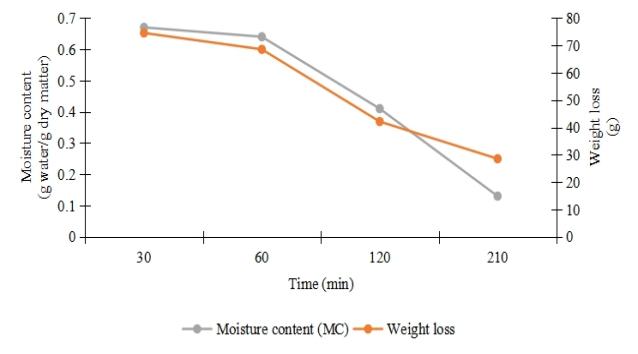

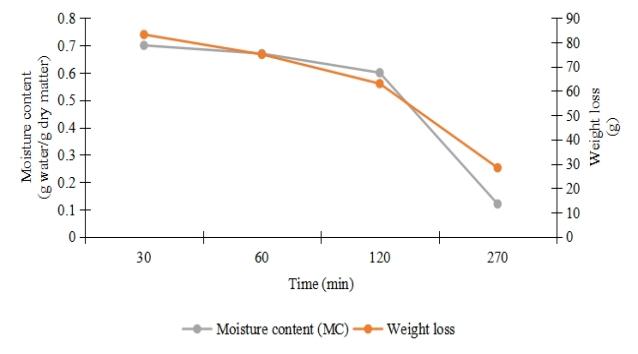

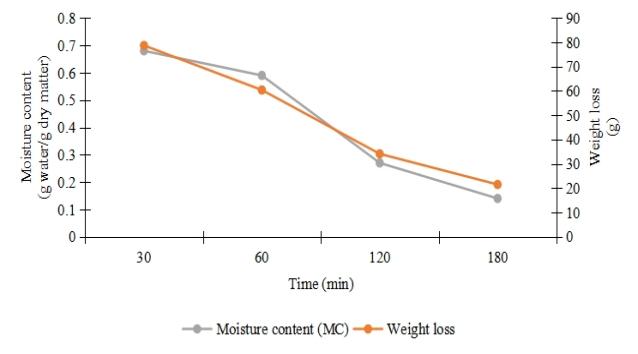

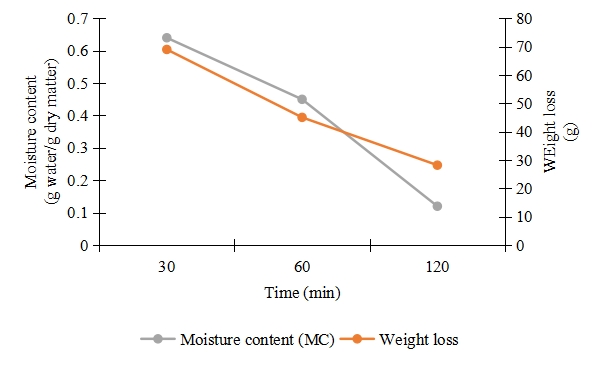


a

b

c

d

e

f

**Figure S2** Moisture content and weight loss depending on the drying time and drying temperature (a: 60 ℃; b: 80 ℃; c: 100 ℃; d: 120 ℃; e: 140 ℃ and f: 160 ℃) for ‘Super Hot’ chili.

Abundance

Time (min)

60 ℃

80 ℃

100 ℃

120 ℃

160 ℃

140 ℃

Capsaicin

Dihydrocapsaicin

Capsaicin

Dihydrocapsaicin

Capsaicin

Dihydrocapsaicin

Capsaicin

Dihydrocapsaicin

Capsaicin

Dihydrocapsaicin

Capsaicin

Dihydrocapsaicin

**Figure S3** Gas chromatography-mass spectrometry (GC-MS) chromatogram of capsaicin and dihydrocapsaicin in ‘Super Hot’ chili extracts at different drying temperatures.


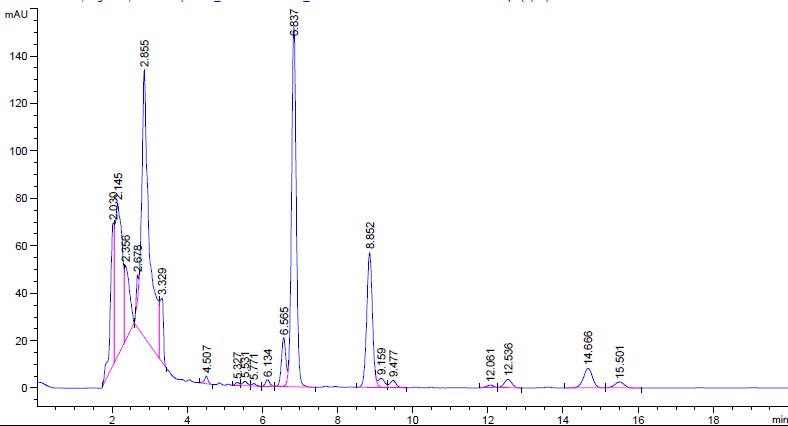

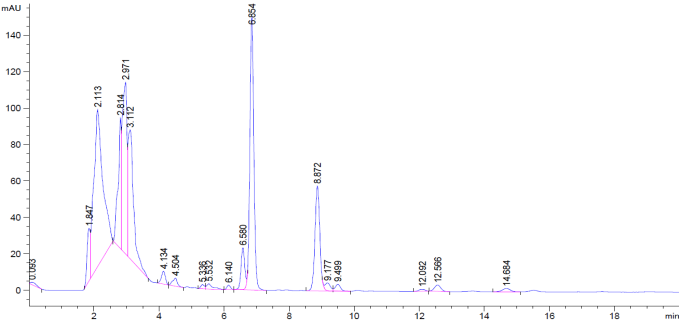

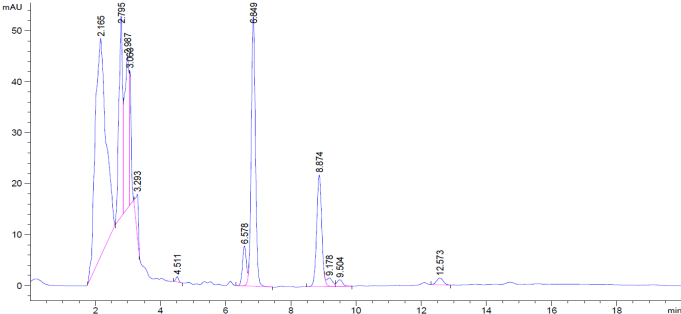

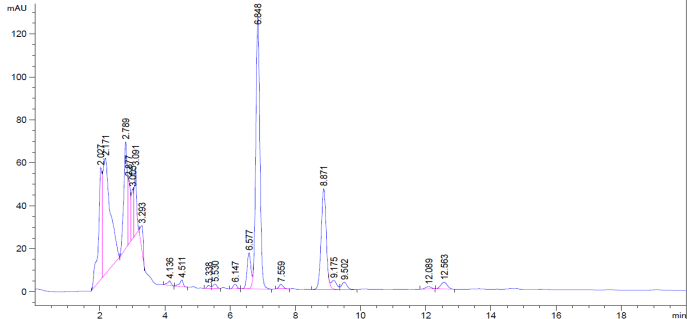

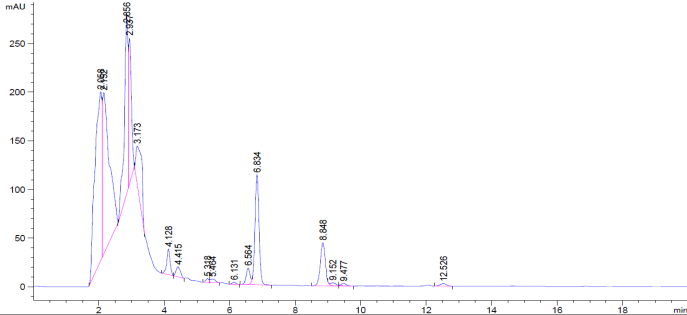

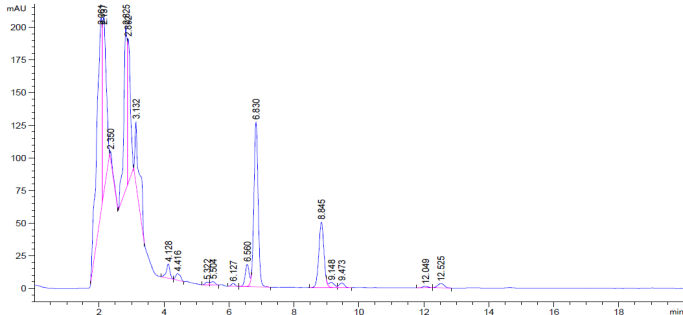


60 ℃

80 ℃

100 ℃

120 ℃

140 ℃

160 ℃

Capsaicin

Dihydrocapsaicin

Capsaicin

Dihydrocapsaicin

Capsaicin

Dihydrocapsaicin

Capsaicin

Dihydrocapsaicin

Capsaicin

Dihydrocapsaicin

Capsaicin

Dihydrocapsaicin

**Figure S4** High performance liquid chromatography (HPLC) chromatogram of capsaicin and dihydrocapsaicin in ‘Super Hot’ chili extracts at different drying temperatures.
